# In-hospital events and resource utilization associated with percutaneous-based vs. open lumbar fusion, stratified by admission status: a national inpatient sample analysis

**DOI:** 10.3389/fmed.2026.1906068

**Published:** 2026-10-07

**Authors:** Hao Yang, Jianlong Li, Yu Sun

**Affiliations:** 1 Department of Spine Orthopedics, Zhumadian Central Hospital, Zhumadian, China; 2 Department of Orthopedics, Nanfang Hospital, Southern Medical University, Guangzhou, China

**Keywords:** elective surgery, in-hospital events, lumbar fusion, national inpatient sample, nonelective admission, percutaneous approach

## Abstract

Minimally invasive surgery (MIS) may be associated with a more favorable short-term inpatient profile after lumbar fusion, but whether these associations differ by admission status remains unclear. In this retrospective observational analysis of repeated cross-sectional 2016–2022 National Inpatient Sample (NIS) discharge data, hospitalizations of adults undergoing lumbar fusion were identified and stratified as elective or non-elective. The exposure was defined by the International Classification of Diseases, Tenth Revision, Procedure Coding System-coded approach as percutaneous-based (percutaneous/percutaneous-endoscopic) vs. open fusion. Survey-weighted multivariable logistic regression accounted for the NIS’s complex design and adjusted for demographics, hospital characteristics, fusion extent, indication/case-mix, and individual Elixhauser comorbidities; formal approach-by-admission interactions were tested. A total of 174,704 hospitalizations met the inclusion criteria. In elective admissions, percutaneous-based fusion was associated with lower odds of blood product transfusion (aOR 0.448, 95% CI 0.311–0.644), acute posthemorrhagic anemia (aOR 0.591, 95% CI 0.475–0.736), and prolonged length of stay (aOR 0.774, 95% CI 0.656–0.914). In non-elective admissions, it was associated with lower odds of acute posthemorrhagic anemia (aOR 0.669, 95% CI 0.491–0.912) and non-home discharge (aOR 0.666, 95% CI 0.502–0.883). Significant approach-by-admission interactions were observed for blood product transfusion (*P* = 0.037) and non-home discharge (*P* = 0.022). These findings indicate that the short-term association profile of percutaneous-based vs. open lumbar fusion differs across admission settings and specific in-hospital measures.

## 1 Introduction

Lumbar disorders are common and disabling conditions that contribute substantially to the global burden of spine-related disability (1). Among the available operative strategies, lumbar fusion remains an accepted and widely used procedure for selected patients with lumbar pathology, particularly in the presence of instability, deformity, recurrent stenosis after prior decompression, or recurrent disc pathology with associated instability (2, 3). In the United States, the use of lumbar fusion has increased substantially over time, accompanied by a marked rise in hospital expenditure, underscoring the growing clinical and economic importance of optimizing perioperative care and inpatient resource use (4). Over the past two decades, minimally invasive spine surgery has been increasingly adopted with the goal of reducing approach-related tissue injury while preserving the therapeutic benefits of fusion (5–7). Prior comparative studies and reviews suggest that, relative to open lumbar fusion, minimally invasive techniques are often associated with less blood loss, shorter length of stay, faster early recovery, and lower perioperative morbidity, while achieving broadly comparable medium- to long-term patient-reported and radiographic measures in appropriately selected patients (8–11). At the same time, the short-term benefits of minimally invasive lumbar fusion may not be uniform across all patient populations and clinical settings, and may vary according to patient comorbidity burden, obesity, and case characteristics (12, 13).

Admission status may be one such clinically important modifier of short-term inpatient risk and early postoperative recovery. In spine surgery more broadly, non-elective fusion cases have been associated with longer length of stay, higher non-home discharge rates, increased readmissions, and greater overall healthcare utilization (14, 15). More specifically, prior lumbar fusion studies have shown that non-elective admission is associated with higher short-term complication rates, including venous thromboembolic events, surgical site infection, wound dehiscence, prolonged hospitalization, and increased hospital charges (15).

These observations raise the possibility that the short-term in-hospital advantages generally associated with minimally invasive lumbar fusion may be attenuated in non-elective admissions, a question that remains insufficiently studied. Prior studies have compared short-term perioperative measures between minimally invasive surgery (MIS) and open lumbar fusion (6, 10). However, whether the short-term in-hospital profile of MIS remains similar across different admission contexts has not been well characterized in large national inpatient datasets. Therefore, using the National Inpatient Sample (NIS), this study compared in-hospital events and utilization measures between International Classification of Diseases, Tenth Revision, Procedure Coding System (ICD-10-PCS)-coded percutaneous-based and open lumbar fusion after stratification by admission status. We additionally performed formal approach-by-admission interaction tests and hypothesized that the magnitude of selected short-term associations would differ by admission status.

## 2 Materials and methods

### 2.1 Data source

This repeated cross-sectional study used hospitalization records from the 2016–2022 NIS, part of the Healthcare Cost and Utilization Project (HCUP) sponsored by the Agency for Healthcare Research and Quality, which represents a stratified 20% sample of U.S. community-hospital discharges. The NIS is an all-payer inpatient database available through the HCUP subject to data purchase and the HCUP Data Use Agreement and provides discharge-level information on patient demographics, hospital characteristics, diagnoses, procedures, discharge disposition, length of stay, and total hospital charges. The files were accessed by an authorized institutional data user after completion of the required HCUP Data Use Agreement training. Because the NIS contains de-identified discharge-level data and involves no direct participant contact, this study was exempt from institutional review board review according to local institutional policy, and informed consent was not required.

### 2.2 Study population

Hospitalizations involving lumbar fusion were identified using prespecified ICD-10-PCS procedure code prefixes according to surgical approach. Open lumbar fusion was identified using the open approach code prefixes (0SG00 and 0SG10), whereas percutaneous-based lumbar fusion was identified using the corresponding percutaneous (0SG03 and 0SG13) and percutaneous-endoscopic (0SG04 and 0SG14) approach prefixes. Hospitalizations containing both open and non-open target fusion approaches were excluded. These PCS approach families have been used to identify lumbar fusion in a prior NIS study (16). Hospitalizations were excluded if the patient was younger than 18 years or if data for age, sex, elective admission status, length of stay, total hospital charges, in-hospital mortality, or required NIS survey-design variables were missing. Fusion extent was represented using the PCS body-part characteristic as one lumbar joint vs. two or more lumbar joints.

### 2.3 Exposure and admission status

The exposure of interest was surgical approach, classified as percutaneous-based lumbar fusion or open lumbar fusion according to ICD-10-PCS coding definitions. Percutaneous-based fusion combined percutaneous and percutaneous-endoscopic approaches. Eligible hospitalizations were further stratified using the NIS ELECTIVE variable, with ELECTIVE=1 classified as elective and ELECTIVE=0 classified as non-elective. The NIS ELECTIVE indicator reflects whether the admission was scheduled in advance; consequently, the non-elective category encompasses a heterogeneous range of unscheduled admission contexts. Analyses were conducted separately within these two groups, and pooled models included a formal approach-by-admission interaction term.

### 2.4 Baseline characteristics

Baseline characteristics included age, sex, race, primary payer, hospital bed size, hospital teaching status, hospital region, fusion extent, clinical indication/case-mix, and Elixhauser comorbidities. Indication/case-mix variables included degenerative disease, trauma, infection, tumor, deformity, and revision-related diagnoses/procedures. The predefined Elixhauser comorbidities included acquired immunodeficiency syndrome (AIDS), alcohol abuse, deficiency anemia, arthritis/collagen vascular disease, chronic blood loss anemia, chronic pulmonary disease, coagulopathy, depression, uncomplicated diabetes, complicated diabetes, drug abuse, heart failure, hypertension, hypothyroidism, liver disease, lymphoma, fluid and electrolyte disorders, metastatic cancer, other neurological disorders, obesity, paralysis, peripheral vascular disorders, psychoses, pulmonary circulation disorders, renal failure, solid tumor without metastasis, peptic ulcer disease, valvular disease, and weight loss.

### 2.5 In-hospital events and utilization measures

The prespecified study measures included selected adverse in-hospital events, in-hospital mortality, non-home discharge, prolonged length of stay, and high total hospital charges. The selected in-hospital adverse events were blood product transfusion, acute lower extremity deep vein thrombosis, postprocedural/surgical site infection, delirium, sepsis, urinary tract infection, acute kidney injury, pneumonia, and acute posthemorrhagic anemia. Full ICD-10-CM/PCS definitions are provided in Supplementary Table S1. Non-home discharge was defined as discharge to a facility or institution other than home, including short-term hospital or other facility transfer; home healthcare was retained in the home category. Hospitalizations ending in in-hospital death or with other or unclassified discharge dispositions were excluded from the non-home-discharge analysis. Prolonged length of stay and high total hospital charges were defined as values above the survey-weighted 75th percentile within each admission stratum, consistent with a prior NIS spine study using upper-quartile thresholds for resource utilization (17). Hospital charges were inflation-adjusted to constant 2022 U.S. dollars using annual Consumer Price Index for All Urban Consumers (CPI-U) values. Length of stay and inflation-adjusted charges were also analyzed as continuous measures.

### 2.6 Statistical analysis

All primary analyses incorporated the NIS’s complex survey design using DISCWT as the discharge weight, NIS_STRATUM as the sampling stratum, and HOSP_NIS as the clustering unit; year-specific stratum and hospital identifiers were used when pooling annual files. Survey-weighted baseline characteristics and standardized mean differences (SMDs) were reported by approach within elective and non-elective admissions. The primary between-approach comparisons were performed using survey-weighted multivariable logistic regression, incorporating the NIS’s complex survey design and adjusting for age, sex, race, primary payer, hospital bed size, teaching status, region, year, fusion extent, degenerative disease, trauma, infection, tumor, deformity, revision-related status, and the individual Elixhauser comorbidities. Approach-by-admission interaction terms were fitted in pooled models to formally assess effect modification. Continuous length of stay and charges were evaluated with survey-weighted Gaussian models, including log-transformed charges as a secondary specification. For sparse binary events, Firth-penalized logistic regression was used as a sensitivity analysis. A degenerative-restricted sensitivity analysis retained cases with degenerative disease and excluded trauma, infection, tumor, deformity, or revision-related features. Additional non-elective subgroup interaction analyses examined age ≥65 years, obesity, high comorbidity burden (≥3 Elixhauser conditions), and hospital teaching status. In-hospital mortality was summarized descriptively because events in the percutaneous-based groups were sparse. No adjustment for multiple testing, such as Bonferroni or false discovery rate correction, was applied because the analyzed measures were prespecified and represented clinically distinct domains; interpretation emphasized effect estimates, 95% confidence intervals, formal interaction tests, and consistency across sensitivity analyses. A two-sided *P-*value < 0.05 was considered statistically significant.

## 3 Results

### 3.1 Study population

A total of 186,392 lumbar-fusion hospitalizations were initially identified. After exclusion due to age eligibility, 176,342 remained; 174,704 hospitalizations met the final required-data, approach-classification, and survey-design criteria (Figure 1). The analytic cohort comprised 149,998 elective admissions (148,630 open and 1,368 percutaneous-based) and 24,706 non-elective admissions (24,299 open and 407 percutaneous-based). Among the percutaneous-based cases, the coded approach included both percutaneous and percutaneous-endoscopic procedures.

**Figure 1:**
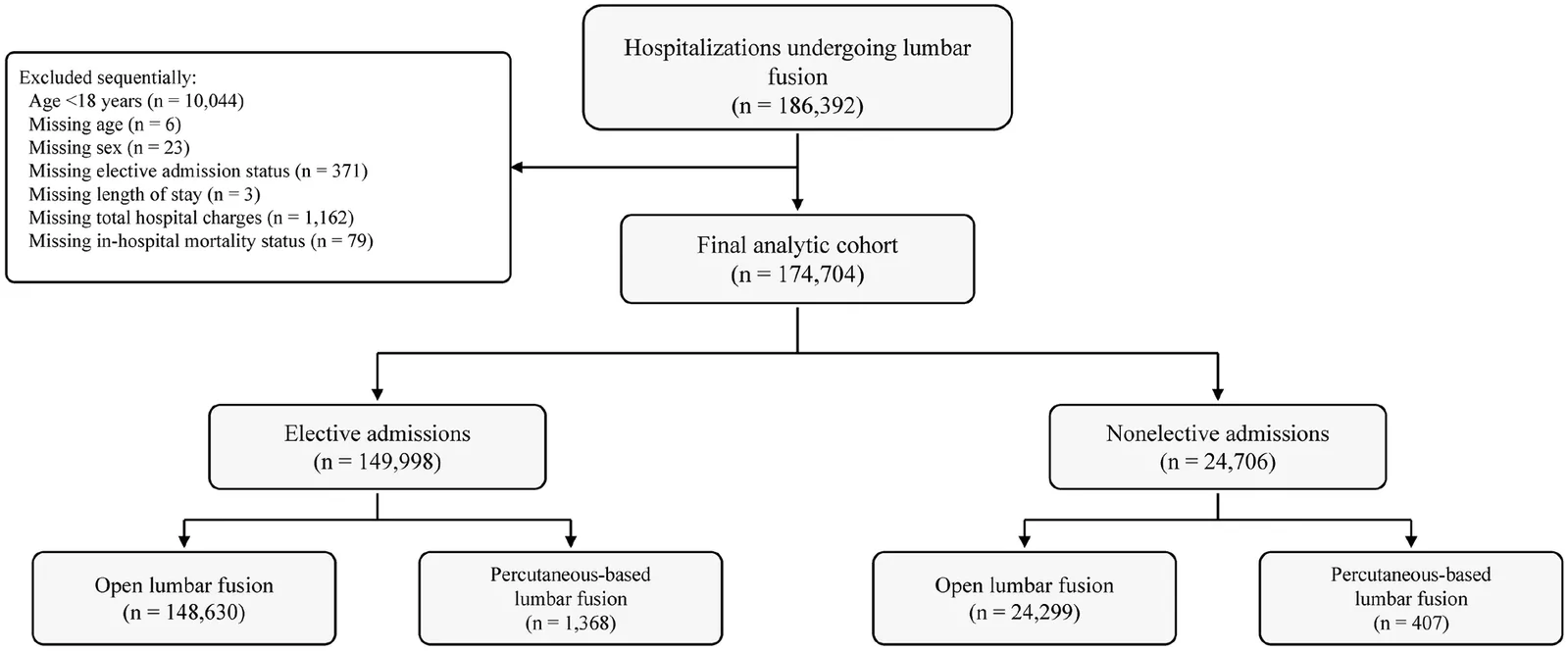
Study flowchart of cohort selection and stratification by admission status and ICD-10-PCS-coded fusion approach.

### 3.2 Baseline characteristics in the elective admission groups

Survey-weighted baseline characteristics in the elective admission groups are summarized in Tables 1, 2. Patients undergoing percutaneous-based fusion were younger than those undergoing open fusion [mean age 61.8 vs. 63.4 years; standardized mean difference (SMD) = 0.129]. Hospital bed size (SMD = 0.194), hospital region (SMD = 0.237), fusion extent (SMD = 0.185), deformity (SMD = 0.109), and revision-related status (SMD = 0.244) showed clinically relevant imbalance before adjustment.

**Table 1:** Survey-weighted baseline demographic, hospital, fusion, and case-mix characteristics by admission status.

Variable	Elective admissions			Non-elective admissions		
	Open (*n* = 148,630)	Percutaneous-based (*n* = 1,368)	SMD	Open (*n* = 24,299)	Percutaneous-based (*n* = 407)	SMD
Demographics						
Age, years, mean (SD)	63.41 (12.37)	61.81 (12.50)	0.129	59.13 (16.82)	59.01 (17.45)	0.007
Female	81,871 (55.1%)	761 (55.6%)	0.011	11,480 (47.2%)	178 (43.7%)	0.071
Race			0.105			0.086
White	118,541 (79.8%)	1,042 (76.2%)		17,462 (71.9%)	280 (68.8%)	
Black	11,637 (7.8%)	141 (10.3%)		2,082 (8.6%)	41 (10.1%)	
Hispanic	8,048 (5.4%)	83 (6.1%)		2,523 (10.4%)	45 (11.1%)	
Asian or Pacific Islander	1,884 (1.3%)	20 (1.5%)		548 (2.3%)	8 (2.0%)	
Native American	636 (0.4%)	10 (0.7%)		193 (0.8%)	5 (1.2%)	
Other	7,884 (5.3%)	72 (5.3%)		1,491 (6.1%)	28 (6.9%)	
Primary payer			0.152			0.078
Medicare	80,155 (53.9%)	670 (49.0%)		11,222 (46.2%)	184 (45.2%)	
Medicaid	9,067 (6.1%)	129 (9.4%)		2,994 (12.3%)	53 (13.0%)	
Private insurance	49,291 (33.2%)	475 (34.7%)		7,442 (30.6%)	117 (28.7%)	
Self-pay	887 (0.6%)	4 (0.3%)		979 (4.0%)	20 (4.9%)	
No charge	68 (0.0%)	0 (0.0%)		75 (0.3%)	2 (0.5%)	
Other payer	9,162 (6.2%)	90 (6.6%)		1,587 (6.5%)	31 (7.6%)	
Hospital characteristics						
Hospital bed size			0.194			0.204
Small	35,850 (24.1%)	240 (17.5%)		3,644 (15.0%)	43 (10.6%)	
Medium	38,351 (25.8%)	448 (32.7%)		6,056 (24.9%)	135 (33.2%)	
Large	74,429 (50.1%)	680 (49.7%)		14,599 (60.1%)	229 (56.3%)	
Hospital region			0.237			0.165
Northeast	26,711 (18.0%)	319 (23.3%)		3,636 (15.0%)	68 (16.7%)	
Midwest	35,325 (23.8%)	283 (20.7%)		4,678 (19.3%)	63 (15.5%)	
South	59,628 (40.1%)	612 (44.7%)		10,924 (45.0%)	208 (51.1%)	
West	26,966 (18.1%)	154 (11.3%)		5,061 (20.8%)	68 (16.7%)	
Teaching hospital	111,754 (75.2%)	1,047 (76.5%)	0.031	19,704 (81.1%)	354 (87.0%)	0.161
Fusion and case mix						
Two or more lumbar joints	56,257 (37.9%)	399 (29.2%)	0.185	11,990 (49.3%)	200 (49.1%)	0.004
Degenerative indication	139,755 (94.0%)	1,293 (94.5%)	0.021	15,632 (64.3%)	208 (51.1%)	0.270
Trauma indication	2,480 (1.7%)	35 (2.6%)	0.062	7,364 (30.3%)	189 (46.4%)	0.336
Infection indication	577 (0.4%)	2 (0.1%)	0.047	1,783 (7.3%)	13 (3.2%)	0.186
Tumor indication	576 (0.4%)	9 (0.7%)	0.037	1,293 (5.3%)	18 (4.4%)	0.042
Deformity indication	22,548 (15.2%)	157 (11.5%)	0.109	3,060 (12.6%)	44 (10.8%)	0.055
Revision-related	28,418 (19.1%)	144 (10.5%)	0.244	3,312 (13.6%)	23 (5.7%)	0.273

Categorical values are unweighted *n* (survey-weighted %) unless otherwise indicated; continuous values are survey-weighted mean (SD). SMD, standardized mean difference.

**Table 2:** Survey-weighted Elixhauser comorbidity characteristics by admission status.

Comorbidity	Elective admissions			Non-elective admissions		
	Open (*n* = 148,630)	Percutaneous-based (*n* = 1,368)	SMD	Open (*n* = 24,299)	Percutaneous-based (*n* = 407)	SMD
AIDS	356 (0.2%)	2 (0.1%)	0.021	109 (0.4%)	1 (0.2%)	0.034
Alcohol abuse	1,821 (1.2%)	19 (1.4%)	0.014	1,260 (5.2%)	20 (4.9%)	0.012
Deficiency anemia	10,962 (7.4%)	94 (6.9%)	0.020	3,550 (14.6%)	67 (16.5%)	0.051
Arthritis/collagen vascular disease	9,361 (6.3%)	77 (5.6%)	0.028	1,585 (6.5%)	30 (7.4%)	0.033
Chronic blood loss anemia	716 (0.5%)	1 (0.1%)	0.078	171 (0.7%)	3 (0.7%)	0.004
Chronic pulmonary disease	27,026 (18.2%)	259 (18.9%)	0.019	3,999 (16.5%)	46 (11.3%)	0.150
Coagulopathy	3,933 (2.6%)	23 (1.7%)	0.066	1,425 (5.9%)	24 (5.9%)	0.001
Depression	26,248 (17.7%)	241 (17.6%)	0.001	3,949 (16.3%)	63 (15.5%)	0.021
Diabetes without complications	21,839 (14.7%)	180 (13.2%)	0.044	2,790 (11.5%)	51 (12.5%)	0.032
Diabetes with chronic complications	13,229 (8.9%)	98 (7.2%)	0.064	3,338 (13.7%)	60 (14.7%)	0.029
Drug abuse	2,216 (1.5%)	23 (1.7%)	0.015	1,344 (5.5%)	19 (4.7%)	0.039
Heart failure	4,923 (3.3%)	44 (3.2%)	0.005	1,592 (6.6%)	26 (6.4%)	0.007
Hypertension	93,796 (63.1%)	810 (59.2%)	0.080	13,853 (57.0%)	232 (57.0%)	<0.001
Hypothyroidism	22,731 (15.3%)	175 (12.8%)	0.072	3,201 (13.2%)	55 (13.5%)	0.010
Liver disease	3,391 (2.3%)	26 (1.9%)	0.027	1,138 (4.7%)	18 (4.4%)	0.013
Lymphoma	417 (0.3%)	3 (0.2%)	0.012	344 (1.4%)	7 (1.7%)	0.024
Fluid and electrolyte disorders	13,962 (9.4%)	88 (6.4%)	0.110	6,394 (26.3%)	115 (28.3%)	0.044
Metastatic cancer	608 (0.4%)	10 (0.7%)	0.043	1,378 (5.7%)	20 (4.9%)	0.034
Other neurological disorders	3,233 (2.2%)	26 (1.9%)	0.019	1,550 (6.4%)	28 (6.9%)	0.020
Obesity	35,214 (23.7%)	320 (23.4%)	0.007	4,874 (20.1%)	79 (19.4%)	0.016
Paralysis	2,878 (1.9%)	31 (2.3%)	0.023	3,190 (13.1%)	36 (8.8%)	0.137
Peripheral vascular disease	5,110 (3.4%)	40 (2.9%)	0.029	978 (4.0%)	13 (3.2%)	0.045
Psychoses	3,946 (2.7%)	39 (2.9%)	0.012	1,057 (4.3%)	15 (3.7%)	0.034
Pulmonary circulation disease	1,009 (0.7%)	10 (0.7%)	0.006	303 (1.2%)	7 (1.7%)	0.039
Renal failure	9,598 (6.5%)	82 (6.0%)	0.019	1,950 (8.0%)	35 (8.6%)	0.021
Solid tumor without metastasis	1,112 (0.7%)	12 (0.9%)	0.014	1,199 (4.9%)	10 (2.5%)	0.132
Peptic ulcer disease	450 (0.3%)	7 (0.5%)	0.033	130 (0.5%)	1 (0.2%)	0.046
Valvular disease	4,123 (2.8%)	45 (3.3%)	0.030	883 (3.6%)	21 (5.2%)	0.074
Weight loss	1,025 (0.7%)	4 (0.3%)	0.057	1,474 (6.1%)	28 (6.9%)	0.033

Categorical values are unweighted *n* (survey-weighted %) unless otherwise indicated; continuous values are survey-weighted mean (SD). SMD, standardized mean difference.

Clinical indication was otherwise broadly similar for degenerative disease (94.5% vs. 94.0%; SMD = 0.021), while percutaneous-based cases had a lower prevalence of two-or-more-joint fusion (29.2% vs. 37.9%) and revision-related coding (10.5% vs. 19.1%). These differences were included in the multivariable adjustment set.

The majority of the individual Elixhauser comorbidities showed small between-approach differences in the elective admission groups. The largest observed imbalances included fluid/electrolyte disorders (6.4% vs. 9.4%; SMD = 0.110) and hypertension (59.2% vs. 63.1%; SMD = 0.080) (Table 2).

### 3.3 Baseline characteristics in the non-elective admission groups

In the non-elective admission groups, age and sex distributions were similar across approaches, but important differences were present in hospital and case-mix characteristics (Tables 1, 2). Percutaneous-based procedures were more frequently performed at teaching hospitals (87.0% vs. 81.1%; SMD = 0.161) and showed differences in bed size (SMD = 0.204) and region (SMD = 0.165).

The largest imbalances in the non-elective groups involved surgical indication: trauma was more common in the percutaneous-based fusion group (46.4% vs. 30.3%; SMD = 0.336), whereas degenerative disease (51.1% vs. 64.3%; SMD = 0.270), infection (3.2% vs. 7.3%; SMD = 0.186), and revision-related coding (5.7% vs. 13.6%; SMD = 0.273) were less common. Fusion extent was similar (49.1% vs. 49.3% for two or more lumbar joints).

Among individual Elixhauser conditions, notable imbalances included chronic pulmonary disease (11.3% vs. 16.5%; SMD=0.150) and paralysis (8.8% vs. 13.1%; SMD = 0.137). These variables were retained in the adjusted models. Tables 1, 2 display unweighted counts together with survey-weighted percentages.

The observed non-elective case-mix differences supported multivariable adjustment and the additional degenerative-restricted sensitivity analysis.

### 3.4 In-hospital events and utilization measures in the elective admission groups

In the elective admission groups, the survey-weighted median length of stay was 2 days for percutaneous-based fusion and 3 days for open fusion; mean length of stay was 2.95 and 3.55 days, respectively (Table 3). Median inflation-adjusted charges were $122,724 and $129,681 in constant 2022 U.S. dollars, respectively.

**Table 3:** Selected survey-weighted in-hospital events and utilization measures by admission status.

Panel A. Selected survey-weighted descriptive measures				
Measure	Elective admissions		Non-elective admissions	
	Open	Percutaneous-based	Open	Percutaneous-based
Continuous utilization measures				
Length of stay, days	3.55 (3)	2.95 (2)	9.12 (7)	9.45 (6)
Inflation-adjusted hospital charges, 2022 USD	$164,967 ($129,681)	$151,341 ($122,724)	$263,122 ($203,905)	$273,645 ($228,237)
Selected categorical measures				
In-hospital mortality	181/148,630 (0.1%)	2/1,368 (0.1%)	208/24,299 (0.9%)	4/407 (1.0%)
Blood product transfusion	8,388/148,630 (5.6%)	30/1,368 (2.2%)	2,529/24,299 (10.4%)	34/407 (8.4%)
Acute posthemorrhagic anemia	23,973/148,630 (16.1%)	121/1,368 (8.8%)	6,196/24,299 (25.5%)	82/407 (20.1%)
Non-home discharge	27,200/148,310 (18.3%)	226/1,365 (16.6%)	10,732/23,979 (44.8%)	163/402 (40.5%)
Prolonged LOS (>75th percentile)	31,918/148,630 (21.5%)	224/1,368 (16.4%)	5,645/24,299 (23.2%)	100/407 (24.6%)
High charges (>75th percentile)	37,182/148,630 (25.0%)	317/1,368 (23.2%)	6,058/24,299 (24.9%)	118/407 (29.0%)

**Table:** 

Panel B. Adjusted continuous utilization analyses		
Measure	Elective admissions Adjusted effect (95% CI); *P*	Non-elective admissions Adjusted effect (95% CI); *P*
Continuous utilization measures		
Length of stay, days	MD −0.469 days (−0.603 to −0.335); <0.001	MD −0.138 days (−1.478 to 1.202); 0.840
Inflation-adjusted hospital charges, 2022 USD	GMR 0.982 (0.933–1.035); 0.506	GMR 1.026 (0.921–1.143); 0.643

Panel A presents selected descriptive measures highlighted in the main text. Continuous values are shown as survey-weighted mean (median); categorical values are shown as unweighted n/N (survey-weighted %). Panel B presents adjusted continuous effects from the primary survey-weighted models: mean difference (MD) for length of stay and geometric mean ratio (GMR) for inflation-adjusted charges. Adjusted associations for all prespecified binary events and formal approach-by-admission interaction *P* values are presented in Figure 2.

Blood product transfusion occurred in 2.2% of percutaneous-based vs. 5.6% of open admissions, and acute posthemorrhagic anemia in 8.8% vs. 16.1%. Prolonged length of stay occurred in 16.4% vs. 21.5% (Table 3).

### 3.5 In-hospital events and utilization measures in the non-elective admission groups

In the non-elective admission groups, the survey-weighted median length of stay was 6 days for percutaneous-based fusion and 7 days for open fusion, while the mean length of stay was 9.45 and 9.12 days, respectively. Median inflation-adjusted charges were $228,237 and $203,905, respectively (Table 3).

Blood product transfusion occurred in 8.4% of percutaneous-based vs. 10.4% of open admissions, and acute posthemorrhagic anemia in 20.1% vs. 25.5%. Non-home discharge occurred in 40.5% vs. 44.8%, whereas prolonged length of stay and high-charge rates were similar in descriptive comparisons (Table 3).

### 3.6 Adjusted associations, formal interactions, and sensitivity analyses

After survey-weighted multivariable adjustment, percutaneous-based fusion in elective admissions was associated with lower odds of blood product transfusion [adjusted odds ratio (aOR) 0.448, 95% CI 0.311–0.644; *P* < 0.001], acute posthemorrhagic anemia (aOR 0.591, 95% CI 0.475–0.736; *P* < 0.001), and prolonged length of stay (aOR 0.774, 95% CI 0.656–0.914; *P* = 0.003). In non-elective admissions, percutaneous-based fusion was associated with lower odds of acute posthemorrhagic anemia (aOR 0.669, 95% CI 0.491–0.912; *P* = 0.011) and non-home discharge (aOR 0.666, 95% CI 0.502–0.883; *P* = 0.005) (Figure 2).

**Figure 2:**
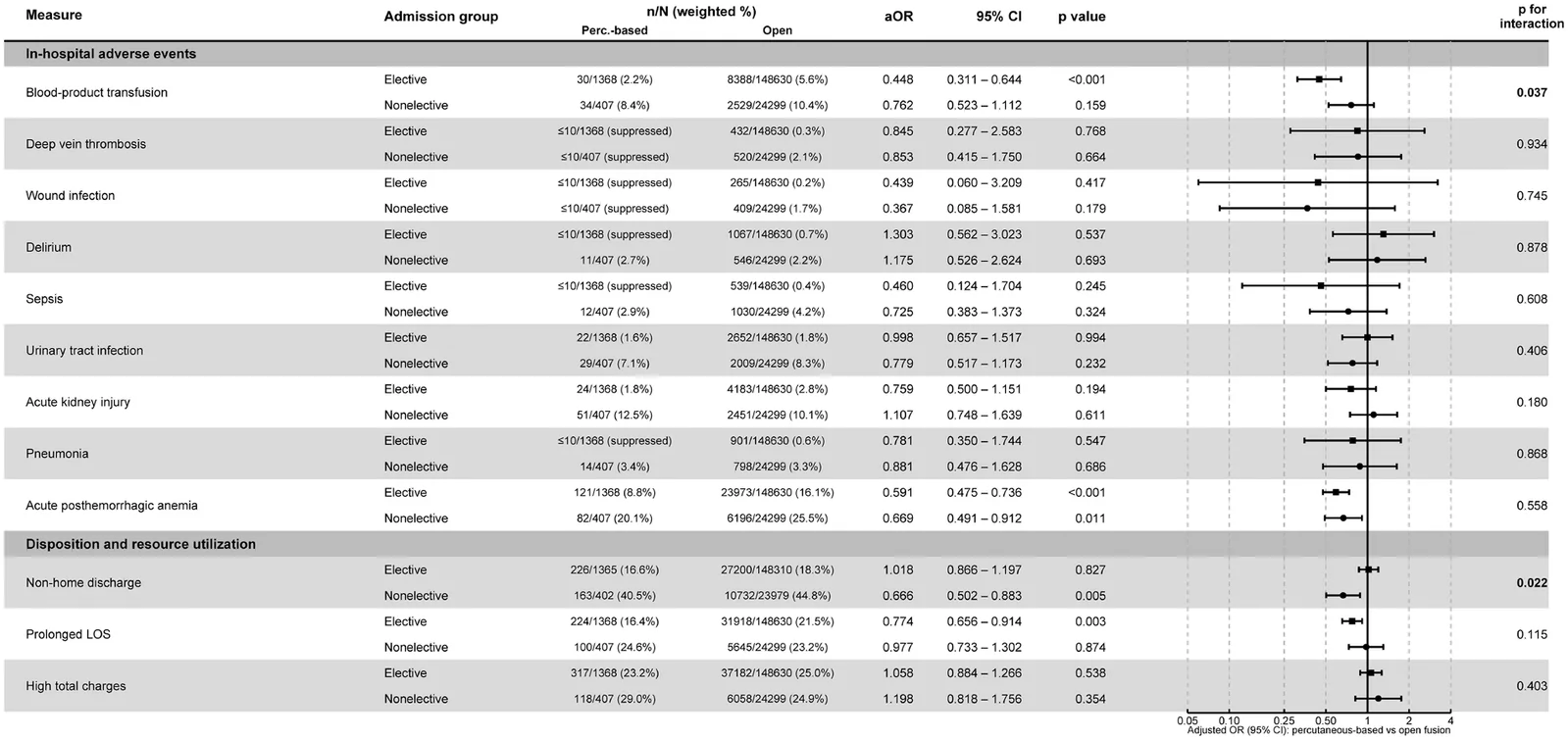
Survey-weighted multivariable logistic regression comparing percutaneous-based vs. open lumbar fusion, stratified by admission status. Squares indicate elective admissions and circles indicate non-elective admissions. For binary measures, numerators and denominators are unweighted analytic counts (*n*/*N*), whereas percentages, adjusted odds ratios, and interaction tests are survey-weighted. *P*-values for the formal approach-by-admission interaction are displayed for each measure.

Formal interaction testing demonstrated significant effect modification by admission status for blood product transfusion (interaction OR 1.745, 95% CI 1.033–2.948; *P* for interaction = 0.037) and non-home discharge (interaction OR 0.673, 95% CI 0.479–0.944; *P* for interaction = 0.022). The reduction in transfusion was more pronounced in elective admissions, whereas the lower odds of non-home discharge were concentrated in non-elective admissions. Acute posthemorrhagic anemia was reduced in both admission strata. In continuous-measure analyses, elective percutaneous-based fusion was associated with a 0.469-day shorter adjusted mean length of stay (95% CI −0.603 to −0.335; *P* < 0.001). The principal elective associations with transfusion, acute posthemorrhagic anemia, and prolonged length of stay remained consistent in the degenerative-restricted sensitivity analysis, and the non-home discharge interaction was also maintained (*P* = 0.023). None of the prespecified non-elective subgroup analyses demonstrated significant effect modification by age, obesity, comorbidity burden, or hospital teaching status (Supplementary Table S3).

Firth-penalized sensitivity analyses for sparse binary events did not identify additional significant approach associations.

## 4 Discussion

In this stratified NIS analysis, the pattern of in-hospital associations between percutaneous-based and open lumbar fusion varied by admission status. In elective admissions, percutaneous-based fusion was associated with lower odds of blood product transfusion, acute posthemorrhagic anemia, and prolonged length of stay. In non-elective admissions, it was associated with lower odds of acute posthemorrhagic anemia and non-home discharge. Formal interaction testing further demonstrated that admission status modified the associations with blood product transfusion and non-home discharge. Taken together, these findings indicate that the short-term association profile of percutaneous-based vs. open fusion differs across admission settings and measured domains. This overall pattern is broadly consistent with the contemporary comparative literature, in which the most reproducible advantages of minimally invasive lumbar fusion are concentrated in short-term perioperative measures such as blood loss and length of stay, while longer-term differences relative to open fusion are often less pronounced (10, 18, 19).

In the elective admission group, percutaneous-based fusion was associated with lower odds of blood product transfusion and acute posthemorrhagic anemia, as well as a shorter length of stay. This pattern is clinically plausible and internally consistent. Transfusion, acute posthemorrhagic anemia, and length of stay are more directly linked to operative exposure and blood loss than many postoperative medical complications (11). Acute posthemorrhagic anemia was lower with percutaneous-based fusion in both admission strata, whereas the significant interaction for blood product transfusion indicated a stronger association in elective admissions. These results therefore fit well with prior prospective, matched, and registry-based studies showing that minimally invasive lumbar fusion is associated with lower blood loss and shorter hospitalization, while long-term measures such as disability, pain, quality of life, and reoperation rates are often similar between approaches (11, 18, 19).

The findings in the non-elective admission groups warrant separate interpretation because the association pattern differed materially from that observed in the elective admissions. Prior work has shown that non-elective spinal fusion is associated with longer length of stay, more frequent non-home discharge, greater readmission burden, and higher financial utilization than elective fusion (14). In-hospital mortality was substantially higher in the non-elective than elective admissions overall; mortality was reported descriptively because of the low event count in the percutaneous-based group. In the present study, the non-elective percutaneous-based group showed lower odds of acute posthemorrhagic anemia and non-home discharge. The lower odds of non-home discharge may reflect an early recovery advantage, potentially related to less tissue trauma, lower early postoperative pain burden, and earlier mobilization (7, 20, 21). The significant interaction for non-home discharge, which persisted in the degenerative-restricted analysis, suggests that this association differed by admission setting. Differences between these national estimates and smaller institution-specific urgent or emergent spine-surgery series may reflect differences in sample size, administrative coding frameworks, non-elective case mix, and the extent of confounder adjustment (14).

Hospital charge measures were broadly comparable between percutaneous-based and open fusion after adjustment for inflation, patient and hospital characteristics, and the NIS’s survey design. Prior studies have demonstrated substantial variation in spinal fusion expenditure across hospitals and procedural settings (22, 23), underscoring that inpatient financial burden reflects multiple components of care beyond surgical approach alone.

These findings have direct clinical implications. In elective lumbar fusion, percutaneous-based fusion was associated with lower odds of transfusion, acute posthemorrhagic anemia, and prolonged length of stay, indicating a favorable short-term inpatient profile in terms of perioperative blood loss and recovery. In non-elective admissions, the association pattern shifted toward lower odds of acute posthemorrhagic anemia and non-home discharge, suggesting that the favorable short-term associations of the percutaneous-based approach are expressed differently within non-elective admission settings. The significant interactions for transfusion and non-home discharge further support admission status as an important modifier of the association between surgical approach and in-hospital events. Accordingly, admission context should be incorporated when interpreting the expected short-term inpatient profile of percutaneous-based vs. open lumbar fusion.

This study has several strengths. It used a contemporary national inpatient database and analyzed elective and non-elective admissions separately rather than combining them into a single analytic population. This design allowed for a more clinically interpretable assessment of how admission context relates to the observed short-term profile of percutaneous-based vs. open fusion. National estimates incorporated the NIS discharge weights, strata, and hospital clustering; the models also formally tested approach-by-admission interactions and additionally adjusted for fusion extent and major indication/case-mix categories. Sensitivity analyses addressed sparse events and restricted the cohort to lower-heterogeneity degenerative cases.

However, several limitations should be considered. First, the NIS is a discharge-level administrative database and does not provide longitudinal follow-up beyond the index hospitalization. Accordingly, this analysis was limited to discharge-level in-hospital events, discharge disposition, and utilization measures recorded during the index hospitalization and could not assess the post-discharge clinical course. Second, surgical approach, diagnoses, and in-hospital events were identified using ICD-10 coding, while detailed clinical and operative factors such as disease severity, American Society of Anesthesiologists (ASA) class, blood loss, operative time, and implant characteristics were unavailable. Because ICD-10-PCS approach coding does not necessarily capture the full spectrum of clinically defined minimally invasive techniques, some clinically minimally invasive procedures may be coded within the open category; such misclassification could attenuate observed between-approach differences, although its exact direction cannot be determined from the NIS. Residual confounding may also remain because unmeasured illness severity and operative complexity can influence both approach selection and events. Third, several events had low event counts, particularly in the non-elective percutaneous-based admissions, and were therefore additionally assessed using sparse-event methods. Finally, no Bonferroni or false discovery rate correction was applied across the prespecified study measures; interpretation therefore emphasized effect estimates, confidence intervals, formal interaction testing, and consistency across sensitivity analyses rather than isolated *P*-values.

## 5 Conclusion

In this national inpatient analysis, the pattern of in-hospital associations between percutaneous-based and open lumbar fusion varied by admission status. In elective admissions, percutaneous-based fusion was associated with lower odds of blood product transfusion, acute posthemorrhagic anemia, and prolonged length of stay. Among non-elective admissions, it was associated with lower odds of acute posthemorrhagic anemia and non-home discharge. Formal interaction testing identified significant differences by admission status for blood product transfusion and non-home discharge. Overall, the association profile of percutaneous-based vs. open fusion differed according to both the specific in-hospital measure and admission status.

## Data Availability

The data analyzed in this study are subject to the following licenses/restrictions: the data that support the findings of this study are available from the Healthcare Cost and Utilization Project (HCUP) National Inpatient Sample. Restrictions apply to the availability of these data, which were used under license for the current study and are therefore not publicly redistributed by the authors. Data may be obtained from HCUP by eligible researchers through the standard application and data use agreement procedures. Requests to access these datasets should be directed to the HCUP.
