# Supplementary material for: In-hospital events and resource utilization associated with percutaneous-based vs. open lumbar fusion, stratified by admission status: a national inpatient sample analysis
Source: Front Med (Lausanne). 2026 Oct 7;13:1906068. doi: 10.3389/fmed.2026.1906068 (PMC13683617; doi:10.3389/fmed.2026.1906068)
Supplement: Supplementary file 1 [file Table1.docx]

**Supplementary Table 1. Summary of reported cases with strictly defined intraspinal tophaceous gout (n = 33)**

| **No** | **Author/**  **Year** | **Age/**  **Sex** | **Level** | **Location** | **Preoperative Misdiagnosis** | **Diagnostic Method** | **Surgical Approach** | **Postoperative Management** | **DECT** | **Follow-up** |
| --- | --- | --- | --- | --- | --- | --- | --- | --- | --- | --- |
| 1 | Wu Z / 2022  [6] | 48/M | L2–L3, L4 | Posterior epidural space | Free disc fragment; epidural tumor | Pathology: tophus | Laminectomy + lesion resection | Not mentioned | No | Complete relief of lumbocrural pain |
| 2 | Wu Z/ 2022  [6] | 63/M | L4–L5 | Left posterior epidural space | Lumbar disc herniation | Pathology: urate crystals | Posterior decompression + fragmentectomy | Not mentioned | No | No recurrence at 3 and 6 months |
| 3 | Si M / 2020  [7] | 66/M | L3–L4 | Posterior epidural space | Abscess or malignant tumor | Pathology: tophus | Epidural mass resection | Not mentioned | No | Steady neurological recovery |
| 4 | Si M / 2020  [7] | 49/F | L3 | Intradural | Intraspinal tumor or disc lesion | Pathology: tophus | Intradural mass resection | Not mentioned | No | Pain disappeared |
| 5 | Chen X / 2020  [8] | 64/M | L4–L5 | Left epidural space | Disc herniation + lateral recess stenosis | Pathology: uric acid crystals | Percutaneous endoscopic decompression | Colchicine + febuxostat | No | No recurrence at 1 year |
| 6 | Chang P / 2023  [9] | 47/F | L4–L5 | Right epidural foramen | Calcified disc herniation or synovial cyst | Pathology: tophus | Decompression + fusion | Not detailed | No | Complete symptom relief at 1 month |
| 7 | Beier CP / 2005  [10] | 29/M | L4–L5 | Epidural with paravertebral extension | Malignant spinal tumor or abscess | Pathology: urate crystals | Hemilaminectomy + decompression | Allopurinol | No | Radiculopathy relieved; persistent back pain |
| 8 | Mishra R / 2020  [11] | 33/M | T10–T11 | Intradural extramedullary | Meningioma | Pathology: tophus | Laminectomy + lesion resection | Referred to rheumatology | No | Independent walking at 3 months |
| 9 | Cao B / 2025  [12] | 22/M | L4–L5 | Intraspinal involving facet joint | Spinal tumor or disc herniation | CT + DECT + pathology | Laminectomy + tophus resection | NSAIDs + febuxostat | Yes | Discharged at 3 months; sensory and motor recovery |
| 10 | Wang K / 2016  [13] | 24/M | T10–T11 | Intraspinal | Not specified | Surgery + pathology | Surgical resection | Not mentioned | No | Symptoms markedly improved |
| 11 | Wang K / 2016  [13] | 44/M | Not specified | Intraspinal | Not specified | DECT + surgery | Surgical resection | Not detailed | Yes | Symptoms markedly improved |
| 12 | Yip CM / 2021  [14] | 29/M | L4–L5 | Posterior epidural space | Not specified | Pathology: tophus | Total laminectomy + mass resection | Colchicine + febuxostat | No | Complete relief of lumbocrural pain |
| 13 | Zhao C / 2025  [15] | 56/M | T8–T9 | Epidural | Spinal cord tumor | MRI + DECT + pathology | Decompression + resection + fixation/fusion | Febuxostat | No | Complete neurological recovery at 10 months |
| 14 | Chen J / 2025  [16] | 16/M | L5–S1 | Epidural | Epidural spinal tumor | CT + MRI + pathology | Decompression + adhesiolysis + resection | Urate-lowering therapy | No | Symptoms completely improved |
| 15 | Gebreselassie KZ / 2023  [17] | 65/M | T4–T5 | Ventral epidural | Malignancy | Pathology: tophus | Minimally invasive laminectomy + biopsy | Allopurinol + colchicine | No | Permanent neurological deficit |
| 16 | Liu SZ / 2024  [18] | 35/M | L3 | Intraspinal and left paravertebral | Spinal tumor | CT + MRI + bone scan + pathology | Resection + decompression + fixation/fusion | Febuxostat | No | Uneventful recovery |
| 17 | Ye Y / 2023  [19] | 29/M | L5–S1 | Left facet joint, epidural | Infectious spondylodiscitis with epidural abscess | CT + MRI + pathology | Debridement + TLIF | Urate-lowering therapy | No | DECT at 2 years: urate deposition completely resolved |
| 18 | Thompson JW / 2021  [20] | 71/F | C4–C6, L3–L5 | Cervical epidural; lumbar multiple lesions | Metastatic malignancy | Serum uric acid + biopsy | Conservative (no surgery) | Colchicine → allopurinol | No | Hand function improved; minimal mobility improvement |
| 19 | Jegapragasan M / 2014  [21] | 24/M | L3–L5 | Epidural/posterior column | Not specified | Clinical + MRI + pathology | Decompression + tophus resection | Referred to rheumatology | No | Discharged same day; symptoms significantly improved |
| 20 | Sanmillan Blasco JL / 2012  [22] | 71/M | C3–C4 | Intradiscal | Cervical disc herniation | Pathology: tophus | Anterior discectomy + fusion | Not detailed | No | Discharged at 3 days; walking at 6 months |
| 21 | Yen HL / 2002  [23] | 68/M | C3–C5 | Intradiscal | Cervical spondylosis with myelopathy | Pathology: tophus | Anterior discectomy + fusion | Benzbromarone | No | Walking with crutches at 6 months |
| 22 | Magid SK / 1981  [24] | 50/F | T1–T9 | Epidural | Epidural abscess | Pathology: tophus | T1–T9 laminectomy | Allopurinol + colchicine | No | Assisted walking at 4 months |
| 23 | Lu H / 2017  [25] | 68/M | L1–L5 | Epidural/posterior column/disc | Preoperative suspicion of gout | DECT + pathology | Laminectomy + discectomy + TLIF | Not provided | Yes | Not provided |
| 24 | Ribiero da Cunha P / 2018  [26] | 64/M | L3–L4 | Epidural | Spinal meningioma or abscess | Pathology: tophus | Laminectomy + mass resection | Urate-lowering therapy | No | Complete remission at 3 months |
| 25 | Wang W / 2017  [27] | 62/M | L3–L4 | Epidural | Not specified | Pathology: tophus | Laminectomy + curettage + fixation | Not mentioned | No | Walking with brace at day 2; no recurrence |
| 26 | Willner N / 2016  [28] | 54/F | L2 | Intradural | Calcified disc fragment, schwannoma, or dural tumor | Pathology: tophus | Hemilaminectomy | No urate-lowering drugs | No | Radiculopathy relieved at 3 months |
| 27 | Zheng ZF / 2015  [29] | 54/M | T3–T12 | Epidural (ligamentum flavum) | Not specified | Pathology: tophus | T1–T5 laminectomy | Allopurinol | No | Back pain and lower limb numbness improved |
| 28 | Xie L / 2019  [30] | 73/M | C5–C6 | Epidural (ligamentum flavum) | OLF or cervical stenosis | Pathology: tophus | Posterior endoscopic decompression | Cervical collar | No | No recurrence at 3 and 6 months |
| 29 | Popovich T / 2006  [31] | 36/F | T5–T7 | Epidural/posterior column/paravertebral | Infection, lymphoma, metastasis, or myeloma | CT-guided biopsy | Laminectomy + decompression | Allopurinol + colchicine | No | Independent walking at 10 months |
| 30 | Paquette S / 2000  [32] | 56/M | Not specified | Intradural (filum terminale) | Calcified disc fragment, schwannoma, or dural tumor | Pathology: tophus | Two-level laminectomy | No urate-lowering drugs | No | No new symptoms at 6 and 12 weeks |
| 31 | Cheng CW / 2018  [33] | 23/M | C4–T11 | Epidural ventral | Epidural abscess, metastasis, or osteomyelitis | Pathology (with infection) | Multilevel laminotomy + debridement | Prednisone + vancomycin | No | Discharged at day 4 |
| 32 | Koskoff YD / 1953  [34] | 44/M | T9–T11 | Epidural | Spinal tumor | Pathology + chemical analysis | Laminectomy + dural exploration | Low-purine diet + probenecid | No | Symptoms improved |
| 33 | Zhang X / 2025 [35] | 26/M | L4–L5 | Posterior epidural space | None (gouty lumbar spinal stenosis suspected preoperatively) | CT + MRI + pathology (gouty granuloma) | Laminectomy + decompression + fusion with internal fixation + tophus resection | Febuxostat + low-purine diet | No | Complete symptom relief at 3 months; no recurrence |
